# Delayed diagnosis and subsequently increased severity of acute appendicitis (compatible with clinical-pathologic grounds) during the COVID-19 pandemic: an observational case-control study

**DOI:** 10.1186/s12876-021-02024-9

**Published:** 2022-01-11

**Authors:** Amitai Bickel, Samer Ganam, Ibrahim Abu Shakra, Inbal Farkash, Rola Francis, Nour Karra, Fahed Merei, Isaac Cohen, Eli Kakiashvili

**Affiliations:** 1 Department of Surgery A, Galilee Medical Center, 22100 Nahariya, Israel; 2 Faculty of Medicine in the Galilee, Bar-Ilan University, Safed, Israel; 3 Department of Internal Medicine E, The Chaim Sheba Medical Centre, Tel-Hashomer, Ramat Gan, Israel; 4 Department of Pathology, Galilee Medical Center, Nahariya, Israel

**Keywords:** Laparoscopic appendectomy, COVID-19, Acute appendicitis

## Abstract

**Background:**

During a global crisis like the current COVID-19 pandemic, delayed admission to hospital in cases of emergent medical illness may lead to serious adverse consequences. We aimed to determine whether such delayed admission affected the severity of an inflammatory process regarding acute appendicitis, and its convalescence.

**Methods:**

In a retrospective observational cohort case-control study, we analyzed the medical data of 60 patients who were emergently and consecutively admitted to our hospital due to acute appendicitis as established by clinical presentation and imaging modalities, during the period of the COVID-19 pandemic (our study group). We matched a statistically control group consisting of 97 patients who were admitted during a previous 12-month period for the same etiology. All underwent laparoscopic appendectomy. The main study parameters included intraoperative findings (validated by histopathology), duration of abdominal pain prior to admission, hospital stay and postoperative convalescence (reflecting the consequences of delay in diagnosis and surgery).

**Results:**

The mean duration of abdominal pain until surgery was significantly longer in the study group. The rate of advanced appendicitis (suppurative and gangrenous appendicitis as well as peri-appendicular abscess) was greater in the study than in the control group (38.3 vs. 21.6%, 23.3 vs. 16.5%, and 5 vs. 1% respectively), as well as mean hospital stay.

**Conclusions:**

A global crisis like the current viral pandemic may significantly affect emergent admissions to hospital (as in case of acute appendicitis), leading to delayed surgical interventions and its consequences.

## Introduction

Acute appendicitis is considered one of the most common abdominal emergencies.

The etiology is diverse and not always clear, and include mucosal ulceration due to enteric infection, foreign bodies, ischemia, and luminal obstruction. The final common pathway of these factors is invasion of the wall of the appendix by intraluminal bacteria together with typical inflammatory changes [1, 2]. It was already reported that short delay until appendectomy, as well as early antibiotic treatment for acute appendicitis, may postpone surgical intervention for a while [3–7]. However, timing of surgery for appendicitis is of paramount importance as significant delay may lead to progression of the inflammatory process, propagating from acute catarrhal appendicitis to supurative (phlegmonous), gangrenous and perforated appendicitis, and abscess formation, a possibility of peritonitis and sepsis [3–8]. According to large series, the expected distribution of the inflammatory process includes severe inflammatory changes in about 32%, and gangrenous appendicitis in particular in about 13.5% [1, 7, 9–11]. These distributions may vary considerably according to age, gender, and comorbidities. As the differential diagnosis is wide, accurate assessment is important to prevent delayed diagnosis that could be detrimental, especially in the elderly with comorbidities [1, 3, 7, 10]. The preferred and most accurate imaging modality for appendicitis is abdominal computed tomography (CT). This significantly decreases the rate of false positive diagnosis [6, 12]. Ultrasound (US) and magnetic resonance imaging (MRI) are more relevant for diagnostic assessment of children and pregnant women [5].

The present COVID-19 pandemic raised deep concerns in the general population that arrival at a hospital could expose people to the viral disease. Fears of being infected has affected behavior even in the presence of a medical necessity [13, 14].

Recent publications have recently addressed the issue of appendicitis during the viral pandemic [15–27]. Several studies have demonstrated reduced admission rate of acute appendicitis due to preferring conservative care by antibiotic treatment at home for mild inflammation [23–27]. Others have shown increased ratio of advanced inflammatory process during the pandemic due to significant admission delay, most probably due to psychological effect [15–22]. As there is a diversity regarding pathologic nomenclature, and as the clinical and the intra-operative presentation of the appendix are not always in accordance with the pathological appearance, we enrolled this retrospective study to show the effects of delayed admission of emergent cases during the COVID-19 pandemic on the severity of the disease, supported by meticulous validation of the clinical presentation with histopathologic findings [1, 2, 28].

## Methods

We conducted a retrospective observational cohort study of all the patients above 18 years old, who were admitted to the Department of Surgery of Galilee Medical Center, Nahariya, with acute appendicitis that was validated by clinical presentation and imaging modalities (the inclusion criteria). Patients admitted during the main surge of the COVID-19 pandemic (March 1st to June 30th 2020) constituted the study group. The control group consisted of patients who were treated for acute appendicitis during a 12-month period prior to the Corona pandemic (January to December 2019). The study was approved by the institutional Helsinki Committee of the Galilee Medical Center (approval number 0074-20-NHR). In this retrospective file, the approval by the committee included the informed consent (for using all relevant medical information) was obtained from all the relevant participants, without revealing any personal information. The patients’ medical files were meticulously explored for medical and laboratory data (gender, age, associated illnesses, the elapsed time since the start of abdominal pain to surgery, intraoperative findings, the surgical approach, the type and duration of antibiotic treatment, hospital stay, perioperative complications, laboratory, pathological, and imaging analysis).

All the surgical interventions were done through the laparoscopic approach. Every gross presentation of the inflamed appendix was validated intra-operatively by an attending and a senior surgeon. All the histopathology findings of the resected specimen were re-evaluated and validated during the conduction of this research by two senior pathologists, for a better definition of the intra-operative findings in cases of controversy. For example, whenever what was seemed to be a suppurative appendicitis on scene while pathologic examination merely exhibited mild inflammatory changes, the category was changed to mild appendicitis, and vice versa.

### Statistical analysis

We used the IBM software SPSS version 25 for statistical analysis. Descriptive statistics in terms of mean, standard deviation (SD), median, and 25-75 percentiles were performed for all the data parameters. Normal distributions of the quantitative parameters were calculated by the Kolmogorov-Smirnov test. According to the results, we used the parametric t-test or the non-parametric Mann Whitney U test to define differences between the time periods. We used Fisher’s exact test to compare categorical parameters of the two groups. A *p *value less than 0.05 was considered statistically significant.

## Results

During 6-months along the main surges of COVID-19 pandemic period, 60 patients were presented to our department of surgery suffering acute appendicitis. During the 12-months of the control period, 97 patients suffering from acute appendicitis were admitted (Table 1). The mean age of both groups did not differ statistically (*p* = 0.19, Fisher’s exact test), as well as the male to female ratios. The clinical presentation and laboratory analysis were not statistically different regarding both two groups. As we focused on intraoperative findings, we did not perform a statistical analysis regarding the imaging (pre-operative) modalities (abdominal ultrasound and computed tomography). Rather, we used it as an adjunct to clarify the clinical and gross presentation of the inflammatory process The mean duration of abdominal pain until surgical intervention (laparoscopic appendectomy) was 2.56 ± 1.53 days in the study group and 1.71 ± 1.39 days in the control group (*p* = 0.001, 2-tail Fisher’s exact test). The distribution of the gross appearance of the infected appendix, as demonstrated during surgery (and supported by histopathologic analysis) have demonstrated significantly larger rates of phlegmonous (suppurative) appendicitis in the study group, while the proportions of mild (catarrhal) appendicitis were less, as compared with the control group. Regarding the proportions of gangrenous appendicitis and peri-appendicular abscess, it was noticed to be much higher in the study group, although not reaching statistical significance, probably due to relatively small numbers of participants (Table 1). Hospital stay was longer in the study than the control group (3.08 ± 1.57 days vs. 2.7 ± 1.51 days, *p* = 0.07, one-tailed Fisher’s exact test). Post-operative complications were not recorded in both two groups, as well as cases of post-operative mortality. There was no need to convert laparoscopic surgeries to open.

**Table 1:** Demographic and clinical characteristics, and intrabdominal findings of patients who underwent laparoscopic appendectomy for acute appendicitis, during the COVID-19 pandemic (Study Group), and during a 12- months of the Control Group

	Study group; n = 60	Control group; n = 97	*P* value
Age	33.0 ± 15.9	38.4 ± 16.9	0.19
Gender			
Male	50%	54	0.80
Female	50%	46%	
Catarrhal inflammation	23 (38.3%)	59 (60.8%)	**0.008**
Phlegmonous (suppurative)	23 (38.3%)	21 (21.6%)	**0.028**
Gangrenous	14 (23.3%)	16 (16.5%)	0.30
Peri-appendicular abscess	3 (5%)	1 (1.03%)	0.15
Hospital stay (days)	3.08 ± 1.57	2.75 ± 1.51	0.19
Duration of antibiotic treatment (days)	3.56 ± 2.08	3.39 ± 1.85	0.74
Duration of abdominal pain until surgery (days) Median: 25–75%	2.56 ± 1.53	1.71 ± 1.39	**< 0.001**

## Discussion

This case-control study demonstrated the profound psychological effect of a viral crisis (the Corona COVID-19 pandemic) on patients who necessitated advanced medical (surgical) help in the hospital. Specifically, proper treatment (surgical intervention for acute appendicitis) was significantly delayed, as reflected by prolonged pre-admission time period of abdominal pain. This was validated by results in recent studies, including in elderly [3, 7, 10, 29]. We decided to investigate this particular medical condition since the consequence of the delayed admission could have been easily evaluated. It should be stressed that our study subjects were not SARS-CoV-2 infected, a fact that might have been associated with increased mortality [30].

The duration of pain prior to surgery was significantly longer in the COVID-19 group, indicating a delay in arrival to the hospital.

We focused on intraoperative findings and clinical parameters that reflect delay in seeking medical attention [2, 6, 7].

Our assumptions were clearly validated by demonstrating significantly increased ratio of phlegmonous (suppurative) appendicitis in the study group, correlating with duration of pre-admission pain. Gangrenous changes and abscess formation also clearly presented increased (although not significant) rate during the COVID-19 pandemic. In comparison to recent publication regarding this topic, our results were precisely validated and were congruent with histopathology workup [15–25]. For example, we approved suppurative appendicitis only following demonstration of inflammatory infiltrate and micro abscesses all-over the appendix wall [1, 2]. According to a worldwide survey, it was demonstrated that during the COVID-19 pandemic, non-operative management was more common, as well as the rate of advanced appendicitis, compared with the pre-COVID era [31].

The mean length of hospital stay was significantly longer in the COVID-19 group. The findings demonstrate that psychological motives (which may be based on solid evidence regarding the risk of viral infection) may affect (and overcome) medical rationale. The upshot is significant delay in receiving proper medical and surgical care [12, 13].

## Conclusions

Understanding the issue of such viral pandemic regarding acute abdominal pain and appendicitis should help promote proper dissemination of information by conventional and social media networks, thus increasing timely treatment. Ultimately, avoiding such postponements reduces the time patients spend in the hospital, which is the motive behind it.

## Data Availability

All data generated or analyzed during this study are included in this published article, In case someone would like to request the data, he should contact the corresponding author.

## Abbreviations

CT: Computed tomography
US: Ultrasound
MRI: Magnetic resonance imaging
